# Increase in Suicide Rates by Hanging in the Population of Tabasco, Mexico between 2003 and 2012

**DOI:** 10.3390/ijerph13060552

**Published:** 2016-06-01

**Authors:** Mervyn Manuel Hernández-Alvarado, Thelma Beatriz González-Castro, Carlos Alfonso Tovilla-Zárate, Ana Fresán, Isela E. Juárez-Rojop, María Lilia López-Narváez, Mario Villar-Soto, Alma Genis-Mendoza

**Affiliations:** 1 División Académica Multidisciplinaria de Comalcalco, Universidad Juárez Autónoma de Tabasco, Comalcalco, Tabasco 86650, Mexico; dramarilit@hotmail.com; 2 División Académica de Jalpa de Méndez, Universidad Juárez Autónoma de Tabasco, Cunduacán, Tabasco 86205, Mexico; thelma.glez.castro@gmail.com; 3 Subdirección de Investigaciones Clínicas, Instituto Nacional de Psiquiatría Ramón de la Fuente Muñiz, Mexico City 14370, Mexico; fresan@imp.edu.mx; 4 División Académica de Ciencias de la Salud, Universidad Juárez Autónoma de Tabasco, Villahermosa, Tabasco 86150, Mexico; iselajuarezrojop@hotmail.com; 5 Hospital General de Yajalón, Secretaría de Salud, Yajalón, Chiapas 29930, Mexico; lilialopez03@hotmail.com; 6 Hospital de Alta Especialidad “Gustavo A. Rovirosa P”, Villahermosa, Tabasco 86020, Mexico; mariovillarsoto@hotmail.com; 7 Instituto Nacional de Medicina Genómica (INMEGEN), Servicios de Atención Psiquiátrica (SAP), Secretaría de Salud, Mexico city 14610, Mexico; adgenis@inmegen.gob.mx

**Keywords:** Mexican population, completed suicide, suicide rate, suicide trends, suicide methods

## Abstract

***Background:*** Worldwide, the suicide rate is decreasing. To examine changes in the rates of completed suicide in the Mexican population from 2003 to 2012, we analyzed these changes according to: (i) the method of suicide; (ii) age group and (iii) gender. ***Methods:*** The data analyzed were obtained from governmental organizations from the State of Tabasco, Mexico. The data provided 1836 cases of subjects born and residing in Tabasco, who completed suicide in this state. ***Results:*** Suicide by hanging was a common choice of suicide method for Mexicans. The rate of suicide by hanging increased from 5.80 to 6.49 per 100,000 persons between 2003 and 2012, a rate percentage increase of 11.89%. ***Conclusions:*** Hanging was found to be the most common choice of suicide in the Mexican population, probably because the materials required are easily available and the method does not require complicated techniques, especially in the 55–64 age group. Strategies for prevention and intervention should be developed for the Mexican population considering suicide rates by age group and gender.

## 1. Introduction

Suicide is a public health problem worldwide that has received increasing attention in recent years [1,2,3,4]. The estimated global mortality of suicide is 14.5 deaths per 100,000 people per year, which equates to one million deaths per year [5,6,7]. Also, it is well known that suicide occurs in developed countries as frequently as in developing ones. In Mexico, the number of deaths by suicide has increased in the past years, becoming a serious issue [8]. As an example, the literature shows that from 1970 to 2007, suicide increased 275% [9]. Furthermore, suicide has become one of the five principal causes of death to 34-year old individuals and the third leading cause in persons between 15 and 25 years old [8,10,11,12]. Unfortunately, information to explain this rapid increase in suicide rates is scarce, since risk factors are very complex and interwoven.

Tabasco State is located in the south of Mexico with a population of more than two million people. This state shows the highest rate of suicide in the country. Therefore, the aim of this study was to investigate the trends between the selection of suicide methods and completed suicide in Tabasco using the most recent mortality data available (2003–2012).

## 2. Materials and Methods

Data were obtained from the Secretary of Health of the state of Tabasco, Mexico. Data were retrieved from the information contained in investigations conducted by the Office of General Procuration of Justice (Procuraduría General de Justicia, in Spanish) of the state of Tabasco, as they were in charge of determining whether each death was a suicide or a murder, as well as providing the death certificates. The cases without complete information or suicide cases performed outside Tabasco State were excluded (years 2005: 2; 2006: 3, 2007: 1; 2008: 2; 2010: 5 and 2011: 5). We only focused on Tabasco’s data because it has the highest suicide rates in the country and there is detailed information available.

The classification of completed suicide was made according to the ICD (International Classification of Diseases and Related Health Problems) instrument; in particular, we used the codes from X60 to X84. Given the small *n* included in our study, the classification by age groups was made according to the Web-Based Injury Statics Query and Reporting System (WISQARS) to facilitate comparison with other investigations [13].

The 2003–2012 study period was chosen because it has the most completed record from the Secretary of Health. The suicide rate was calculated based on the results from the Population and Housing Census performed by the National Institute of Statistic and Geography (INEGI, initials in Spanish) (2005 and 2010) and main demographic indicators (population projections) provided by the National Council of Population (CONAPO, initials in Spanish). The percentage change in rate was used to quantify changes in suicide rates between 2003 and 2012. They were determined according to reports in the literature as: (rate in 2012 − rate in 2003)/(rate in 2003) × 100% [14]. Also, line charts were plotted to reveal changes in suicide rates over the period studied. Finally, we calculated the rate of suicide by age group, suicide method and gender.

## 3. Results

**Figure 1:**
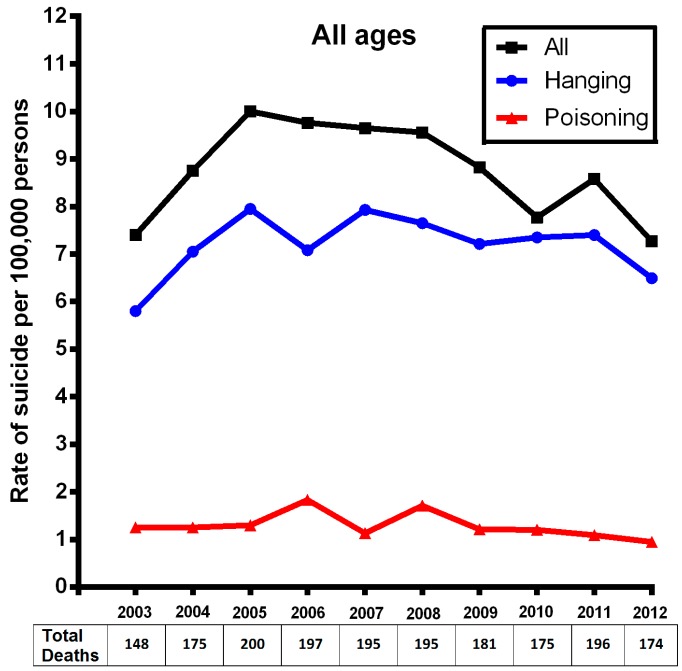
Suicide rate per 100,000 persons including all age groups.

Table 1 shows the changes in suicide rates in the state of Tabasco between 2003 and 2012. In 2012, 178 completed suicides reflected an increase on the overall rate of 1.75% since 2003. The rate varied from 7.40 to 7.53 per 100,000 persons (Figure 1). The main methods used to achieve death were hanging, poisoning and firearms. In 2012, suicides by hanging, poisoning and firearms constituted 99.86% of all suicides in Tabasco. Moreover, suicide by hanging constituted 86.8% of all suicides, which implied an increase since 2003 of 11.83% (hanging represented 78.37% of all suicides in 2003). However, decreases were observed when using poisoning (−24%), drowning and submersion (−100%) and firearms (−68%) as methods to achieve death.

### 3.1. Suicide and Age Group

Comparisons by age groups and methods of suicide are shown in Table 1. The age group with the highest rate of suicides was 15–24 years, although a slight decrease was observed in their overall suicide rate (−5.38%). Between 2003 and 2012, the suicide rate saw a major increase in the 55–64 years group, with a percentage change of 180%. In addition, the percentage change in rate was 15.26% for the 25–34 year old group. In contrast, two groups showed a drop in the percentage change in suicide rate: 0–14 years and 45–54 years.

### 3.2. Suicide and Suicide Method

The analysis of all age groups showed that suicide by hanging from 2003 to 2012 increased 11.89%. Meanwhile, in the 25–34 years group, hanging increased by 33.63%; that is, hanging represented a suicide rate of 84.6% in 2003 and increased to 97.35% in 2012 in this age cohort. Similarly, the suicide rate by hanging in the 25–34 year group increased substantially in 2004 followed by a decrease in the period of 2010 to 2012. Importantly, hanging in the age-group of 55–64 years exhibited a percentage change in suicide rate of 186%; however, the percentage change in rate remained almost unchanged from 2003 (75%) to 2012 (76.78%). Finally, in 2005, the suicide rate by hanging decreased, while poisoning increased.

### 3.3. Suicide by Gender

**Figure 2:**
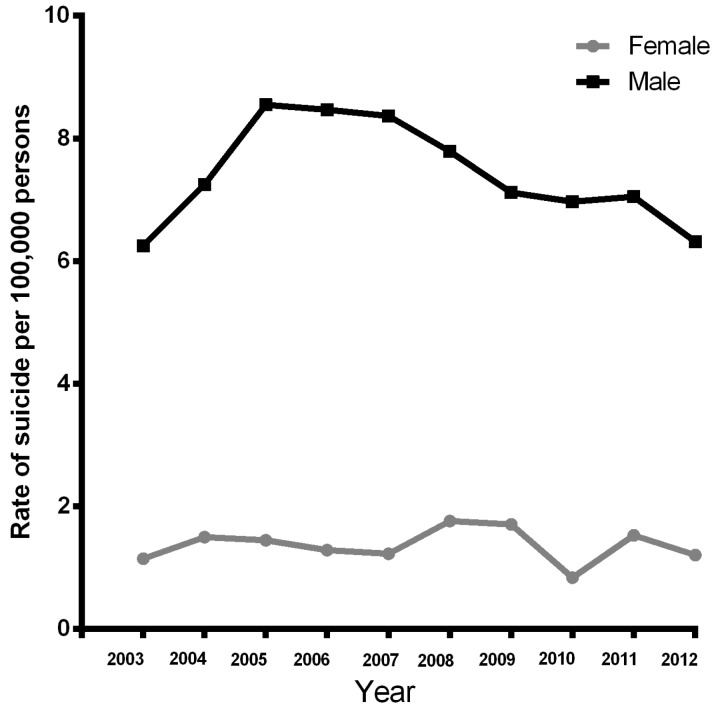
Suicide rate per 100,000 persons by gender, in 2003 and 2012 in Mexican population.

**Figure 3:**
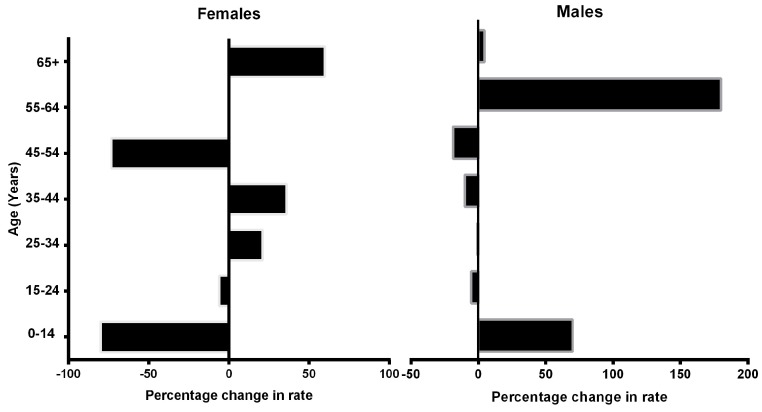
Gender differences of percentage change in rate between 2003 and 2012 by age groups in Mexican population.

In the female group, the change in suicide rate observed was 5.21%, whereas for males it was only 1.21%. Figure 2 shows the changes in suicide rate by gender in the ten years of the analysis. In the female group an increase of 60% was observed in 65 year olds and over. However, a decrease was observed in the 0–14 years of age group (−80.0%), followed by the 45–54 years group (−73.3%). In contrast, males 0–14 years old showed a suicide increase of 70% and those 55–64 years of age showed an increase of 180.0% (Figure 3).

## 4. Discussion

In this study we analyzed the trend of suicide by method used, age and gender in the state of Tabasco, Mexico. Initially, we found that the rate of suicide remained virtually unchanged since 2003. Second, suicide by hanging is the principal method used in our population. In people between 55 and 64 years, suicide by hanging increased 186.66% in the period studied; this increase was principally seen in males.

The literature recently reported that suicide rate has decreased in many countries [15,16]. Fond *et al.* [15] reported a decrease of 46% in Estonia and 26.2% in Rumania, suggesting that preventive actions and prompt medical care can decrease suicide mortality. However, our results show a slight increase of suicide prevalence (1.75%) between 2003 and 2012 in the Tabascan–Mexican population. Our results are consistent with other reports in the literature for the Mexican population that found an increase in the rate of suicide from 4.6 in 2010 to 4.9 in the year 2011 [17], like the ones observed in the Korean population [18].

In our population, there are clear differences by gender. We found a change in suicide rate of 5.21% in males. This result is different from that observed at a national level in males of 36.47% between 2000 and 2013 [17,19]. However, this difference could be explained considering the fact that Tabasco State in Mexico has a historic higher prevalence of suicide than the rest of the country. However, the suicide prevalence remained unchanged in females.

Our analysis by method chosen to achieve death showed that the most frequently used was hanging, representing 86.8% of the suicide rate.

In a study which analyzed the international suicide patterns derived from the WHO mortality database, hanging was the predominant method of suicide in most countries including Mexico [20]. As can be expected, there are deviations from this predominant pattern as other populations have referred jumping [21] and firearms [13] as the most common methods used for suicide. Also, the percentage of the method of hanging observed in our study is higher than in the rest of the country of 75.7% [22]. It is important to emphasize that the suicide methods used in our population are considered more lethal.

On the other hand, we examined the data of suicide victims between 2003 and 2012 by age groups. Our results were in accordance with other reports where completed suicide in the group between 15 and 24 years was most frequent [13,21,23,24,25,26,27] including previous reports on the Mexican popualtion [22]. Another study showed that the 54–65 age group had the highest suicide rate [17,24,25,26]. Similarly in our study, the 25–34 and 55–64 age groups exhibited an increase in suicide by hanging when suicide methods were analyzed. This situation is more severe in males, as we observed that suicide by hanging associated with age (55–64 years) experienced an increase of 180% in men. What is more, the male gender has been more frequently related to suicides due to unemployment [28] as well as alcohol consumption [29]. Perhaps the 180% increase in our male population between 55 and 64 years was the consequence of high unemployment and/or high alcohol consumption in the area; therefore, these risk factors should be evaluated in future studies.

One issue that is important to note in the present manuscript is the dramatic increase in suicide by hanging. This suicide method is largely governed by accessibility, sociocultural norms and acceptability. In terms of accessibility, other traditional methods, such as poisoning or firearms, required more complicated techniques and materials that may be difficult to obtain. While acceptability provides a general framework of beliefs about whether or not to commit suicide and which method to use, sociocultural norms provide a framework for how to proceed. In this way, hanging is a selective method for many subjects as it is violent, requires some preparation and needs some degree of courage [30]. In addition, in most of the cases, hanging is done privately and at times and places that provide limited possibilities of being found before they die, making it an eligible choice for those with more death-oriented intentions [31]. However, we could not exclude the possibility that the suicide by hanging in our population could be by imitation, because it is known that this is a factor that triggers the suicide behavior [32].

We want to emphasize that even though poisoning and firearms are among the most commonly used methods of suicide in Mexican population, a considerable decrease in poisoning (−24%) was observed in 2012, as well as firearms (−68%). The poisoning decrease could be due to the efforts undertaken since 2008 by the Secretary of Health to control drug sales, by only selling them with a medical prescription. Regarding firearms as suicide method, the federal government carried out an enforcement of the Federal Law of Firearms and Explosives about 8 years ago. These measures must be kept in mind since they discourage the use of these suicide methods.

There are limitations in this study that should be mentioned. First, the information used covered 10 years of suicide data in Tabasco, an urban city in the south of Mexico, so it is not possible to make inferences regarding suicides in other geographic regions or time periods other than from 2003 to 2012. Second, the government records may underestimate the number of deaths by suicide because some suicides may be classified as “undetermined” deaths; in our research we considered those undetermined deaths to represent 2% of suicides. Also, we did not take into consideration risk factors such as psychiatric illness, substance abuse or social situations (e.g., economic crisis) that may influence suicide rates. Finally, the data came from one state of Mexico and should not be generalized for the entire Mexican population. To sum up, this study is the first of its kind in dealing with suicide methods in Mexican population; this approach reinforces the importance of scientific research to improve strategies for suicide prevention and education.

## 5. Conclusions

Hanging was found to be the most common choice of suicide in the Mexican–Tabascan population and was related to an increase in the overall suicide rate. Also, we found that in males between 55–64 years of age, suicide increased 180%. In addition, limiting access to poisons and firearms has been an effective strategy to decrease suicide by these particular methods and it is likely that hanging was substituted for these methods. Therefore, we find the most fruitful approach to reducing overall suicide rates is to determine and manage the main risk factors (unemployment, alcohol consumption, mental illness, and others) and to focus on the accessibility of lethal suicide methods such as hanging. Given that the relationship between the availability of suicide methods and the level of suicide is principally mediated by hanging, this suicide method should be the main target for prevention. Due to its high lethality and particular features, such as being performed in private places, interventions must occur at an earlier time. Research into the causes of suicide indicates that a combination of social and personal factors contributes to the emergence of suicidal ideation and future suicide attempts. Health professionals and mental health professionals in particular, should be aware of people’s stressful social circumstances and the probable emergence of depressive symptoms, both clearly related to future suicide attempts. We know that the implications of this study are only one part of the improvement of an effective prevention strategy for suicide.

## Figures and Tables

**Table 1:** Suicide rate per 100,000 persons by age group and suicide method in the state of Tabasco from 2003 to 2012.

Age (Years)	Method	2003 *n* = 148	2012 *n* = 174	Percentage Change in Rate
All	All	7.40	7.53	1.75
	Hanging	5.80	6.49	11.89
	Poisoning	1.25	0.95	−24
	Firearm	0.25	0.08	−68
0–14	All	0.3	0.21	−30
	Hanging	0.25	0.21	−16
	Poisoning	0.05	0	−100
	Firearm	0	0	0
15–24	All	2.6	2.46	−5.38
	Hanging	2.15	2.20	2.32
	Poisoning	0.3	0.25	−16.6
	Firearm	0.1	0.0	−100
25–34	All	1.3	1.51	15.26
	Hanging	1.1	1.47	33.63
	Poisoning	0.15	0.04	−73.3
	Firearm	0	0	0
35–44	All	1.45	1.42	−2.06
	Hanging	1.0	1.08	8
	Poisoning	0.45	0.30	−33.33
	Firearm	0	0.04	
45–54	All	1.05	0.77	−30
	Hanging	0.7	0.64	−14.2
	Poisoning	0.2	0.12	−50
	Firearm	0.1	0	−100
55–64	All	0.2	0.56	180
	Hanging	0.15	0.43	186.66
	Poisoning	0.05	0.08	60
	Firearm	0	0.04	
≥65	All	0.50	0.56	12
	Hanging	0.40	0.40	0
	Poisoning	0.05	0.12	140
	Firearm	0	0	0

Note: Only the most frequent methods were included.

## Abbreviations

The following abbreviations are used in this manuscript:

ICD	International Classification of Diseases and Related Health Problems
WISQARS	Web-Based Injury Statics Query and Reporting System
INEGI	National Institute of Statistics and Geography in Spanish
CONAPO	National Council of Population in Spanish
